# Detection of HPV E6 oncoprotein from urine via a novel immunochromatographic assay

**DOI:** 10.1371/journal.pone.0232105

**Published:** 2020-04-22

**Authors:** Cristina Mendes de Oliveira, Laura W. Musselwhite, Naitielle de Paula Pantano, Fabiana Lima Vazquez, Jennifer S. Smith, Johannes Schweizer, Michael Belmares, Júlio César Possati-Resende, Marcelo de Andrade Vieira, Adhemar Longatto-Filho, José Humberto Tavares Guerreiro Fregnani

**Affiliations:** 1 Molecular Oncology Research Center–Barretos Cancer Hospital, Brazil; 2 Duke Cancer Institute, Duke University, Durham, NC, United States of America; 3 Prevention Department—Barretos Cancer Hospital, Brazil; 4 Gillings School of Global Public Health, University of North Carolina, Chapel Hill, NC, United States of America; 5 Arbor Vita Corporation, Fremont, California, United States of America; 6 Gynecology Department—Barretos Cancer Hospital, Brazil; 7 Medical Laboratory of Medical Investigation (LIM) 14, Department of Pathology, Faculty of Medicine, University of São Paulo, Brazil; 8 Institute of Life and Health Sciences (ICVS), University of Minho, Braga, Portugal; 9 ICVS / 3B's—Associated Laboratory to the Government of Portugal, Braga / Guimarães, Portugal; 10 Teaching and Research Institute—Barretos Cancer Hospital, Brazil; 11 Teaching and Learning Department, A.C. Camargo Cancer Center, Brazil; Rudjer Boskovic Institute, CROATIA

## Abstract

Cervical cancer is a significant public health problem, especially in low- and middle-income countries, where women have little access to cervical cancer screening; consequently 80% of cervical cancer related mortality occurs in these regions. The development of screening methods that need less infrastructure thus represents an urgent medical need. The study aims to compare the detection rates of high-risk human papillomavirus 16 and 18 E6 oncoprotein in urine, vaginal self-collected, and cervical scrapes of women using the Onco***E6***^™^ Cervical Test and compare the HPV16 and/or HPV18 E6 detection rates with the HPV DNA testing. Paired urine, vaginal self-collected and cervical specimens were collected from 124 women who participated in cervical cancer screening or treatment in this proof-of-concept study and underwent to HPV16/18-E6 testing and high-risk HPV DNA testing prior to treatment of cervical neoplasia or cancer. Concordance between urinary, vaginal and cervical HPV16/18-E6 and HPV-DNA testing was evaluated for patients classified as negative group (<CIN2) and histological positive group (CIN2, CIN3 and invasive carcinoma). Overall, HPV16/18-E6 oncoprotein was detected in 30.6% of cervical samples, 20.3% of self-collected vaginal samples and 21% of urine samples. Regarding the clinical sensitivity, the HPV16/18-E6 oncoprotein was not detected in CIN2 cases, and was detected at low rates in CIN3 cases. The clinical sensitivity of the HPV16/18-E6 oncoprotein for detecting invasive cervical cancer was 70% for cervical scrapes, 55% for self-collected vaginal samples and 52% for urine samples. This study reports the urinary detection of E6 oncoprotein *in vivo* for the first time and our results suggest that this detection is only for invasive/microinvasive lesions. Then, further protocol development and standardization to achieve a clinical sensitivity for CIN2/3 detection close to what can be achieved for invasive lesions using the physician collected cervical is needed.

## Introduction

Cervical cancer is the fourth most common cancer in women worldwide, with an estimated 567,847 new cases and 311,365 deaths occurring annually. Approximately 85% of the global burden is registered in less developed regions, highlighting a major public health problem [1]. In Brazil, cervical cancer is the third most frequent cancer in women, with over 16,000 new cases each year [2]. This high incidence of cervical cancer is especially disturbing when considered that cervical cancer develops over many years through precancerous stages, where treatment can be safely and effectively executed. A key challenge is hence the enablement of screening methods that are most appropriate for use in regions of urgent need.

The implementation of organized screening programs based on cervical cytology (Pap test) has led to a significant reduction of cervical cancer incidence and mortality in high-income countries [3–5]. Such reduction, however, has not been achieved in low- and middle-income countries (LMICs). Shortfalls are mainly due to the poor screening program coverage rates, lack of cytology quality control and limited population access to the health care system [6, 7]. Several high-income countries, including the Netherlands [8] and USA [9], are now promoting a paradigm shift in their programs from cervical cytology to high-risk (hr)-HPV testing, which offers increased sensitivity and an improved negative predictive value over cytology [10]. Regardless of the screening method, the success of screening programs depends considerably on a high coverage rate within the target population. It is hence beneficial to not only improve screening test sensitivity and specificity, but to also increase the number of women participating in screening. To improve adherence, particularly of under- and never-screened women, self-collection of vaginal samples has been proposed [11–16]. Some women, however, report pelvic discomfort and/or confusion about how to perform the vaginal self-collection [16]. As an alternative to vaginal self-collection, urine based self-collection has been suggested to be more acceptable by many subjects [17]. Detection of HPV DNA in paired urine and cervical specimens has given inconsistent results with the detection of cervical intraepithelial neoplasia (CIN) 2 or greater (2+), yielding sensitivities ranging from 63 to 95% and specificities of 23 to 89% [18–25]. Recent data derived from use of the Trovagene test has resulted in relatively high sensitivity for high-grade precancerous lesions in higher risk colposcopy referral patients [26, 27]. Urine-based HPV DNA testing remains an attractive alternative to increase screening coverage, mainly among women subgroups that cervical HPV detection is difficult [28].

The presence of HPV E6 oncoprotein is necessary for oncogenic transformation, and its detection in self-collected biological specimens might be an attractive approach in resource-limited settings with a high prevalence of cervical cancer. The detection of the E6 oncoprotein of HPV16/18 from physician-collected cervical samples [29–31] showed promising results in several settings. The objective of this study was to assess the detection of HPV16/18 E6 oncoprotein in urine using the OncoE6 point-of-care test.

## Materials and methods

### Study population

This study was conducted between January and September of 2017 among 124 non-pregnant women, aged 25–64, who attended the Cancer Prevention or the Gynecologic Oncology Departments of Barretos Cancer Hospital (HCB), Brazil. None of the participants had received HPV vaccination, were hysterectomized or had treated an HPV infection before.

Women with invasive cervical cancer were enrolled during the first visit at the outpatient clinic of the Gynecologic Oncology Department, and specimens were collected prior to treatment. Women with histology confirmed CIN2 or 3 were enrolled in the Outpatient Surgical Center, prior to a loop electrosurgical excision (LEEP) procedure, and women with normal cytology were enrolled in the Prevention Department during cervical cancer screening.

The local Research Ethics Committee and the Brazilian National Research Ethics Commission (CONEP) approved the study (CAAE 62057316.8.0000.5437). All participants provided written informed consent, and all the personal information was maintained encrypted in a database to ensure participants’ data confidentiality.

### Specimen collection

Prior to a pelvic examination, women provided a random urine sample in a 80 mL polypropylene container, and a self-collected vaginal sample was obtained via the Viba-Brush® (Rovers Medical Devices, Oss, the Netherlands). Women then underwent pelvic examination by a gynecologist, and a physician-collected cervical scraping was obtained via the Cervex-Brush Combi® (Rovers Medical Devices, Oss, the Netherlands). Self-collected vaginal and physician-collected samples were preserved in a methanol-based liquid medium (CellPreserv, Kolplast, Brazil).

Within two hours of sample collection, specimens were sent to the Molecular Oncology Research Center of the Barretos Cancer Hospital for processing. Five milliliters of each vaginal self- and physician-collected sample were retrieved from the preservative medium to perform the Onco***E6***^™^ Cervical Test and were stored at 4°C. Six milliliters of urine were added to a 50mM solution of EDTA and used to perform HPV DNA testing. The remaining urine was used to perform the Onco***E6***^™^ Cervical Test without solution of EDTA. Self-collected vaginal samples, physician-collected samples and urine samples that were designated for HPV DNA testing were stored at 4°C; urine aliquots designed to conduct the Onco***E6***^™^ Cervical Test were stored at -20°C. Testing for HPV DNA and E6 oncoprotein were performed on each physician collected sample, vaginal self-collected sample and urine self-collected sample.

### HPV tests

For each participant, HPV DNA testing and Onco***E6***^™^ Cervical Test were performed on each of the following specimens: urine sample, self-collected vaginal sample, and physician-collected cervical sample.

Detection of HPV DNA was performed using the Cobas® 4800 HPV platform (Roche, Indianapolis, IN, USA) which is a multiplex real-time PCR assay that provides specific genotyping information for HPV16 and 18, while concurrently detecting 12 other high-risk types (HPV 31, 33, 35, 39, 45, 51, 52, 56, 58, 59, 66 and 68) in a pooled result. Vaginal self-collected and the physician-collected samples were performed according to the manufacturer’s instructions. An aliquot of six milliliters of urine added to a 50mM solution of EDTA was used to perform the HPV DNA testing according to the Cobas® 4800 HPV standard protocol.

The detection of the HPV E6 oncoprotein was performed using the Onco***E6***^™^ Cervical Test (herein referred as to: “HPV16/18-E6 test”; Arbor Vita Corporation, Fremont, CA, USA), an immunochromatographic assay that detects elevated levels of HPV16/18-E6 oncoproteins using a lateral flow format and high-affinity monoclonal antibodies to capture / detect the E6 oncoprotein from cell lysates generated from cervical specimens. Five milliliters each of the self-collected vaginal and the physician-collected cervical samples were centrifuged. The resulting pellet was suspended in 930μL of Rinse Solution and transferred to a test tube, both provided with the Onco***E6***^™^ Cervical Test kit. The E6 test was performed according to the manufacturer’s instructions [32]. Urine specimens were shaken up before aliquots of 7.5mL, 15mL or 30mL were removed and centrifuged. The resulting pellet was also suspended in 930μL of Rinse Solution and then transferred to the test tube. The test was performed according to the manufacturer’s instructions except for the extraction step: upon communication with the manufacturer, volumes for the Lysis Solution and the Conditioning Solution were deceased by 50% with regard to the regular protocol; 416μL and 39μL were used.

### Statistical analysis

Women were divided in two groups: negative (<CIN2: without intraepithelial lesion on cervix or CIN1) or positive (CIN2+: CIN2, CIN 3 and invasive carcinoma) for disease according to the histological analysis of the biopsy or LEEP by pathologists blinded to the clinical exam and medical history. Women with visible cervical lesions observed upon colposcopy underwent biopsy or endocervical curettage as clinically indicated by the gynecologist performing colposcopy. Women without an apparent abnormality did not undergo cervical biopsy and were categorized as negative (<CIN2).

Statistical analyses were performed using R (http://www.R-project.org/), and the significance level was set at 5% for all tests. All reported p-values are two-sided.

The McNemar test [33] and confidence interval were used to compare the positivity rates of the HPV DNA test and the E6 test. The Kappa coefficient was calculated to evaluate the agreement between the tests. For the analysis of clinical accuracy, sensitivity and specificity rates (and respective confidence intervals) were calculated based on the clinical pathological diagnosis.

## Results

The study included a total of 124 women aged 25 to 64 years (median age = 40 years). Of those, 44 (35.5%) had invasive carcinoma, 26 (21.0%) CIN3, 9 (7.2%) CIN2, 2 (1.6%) CIN1 and 43 (34.7%) without precursor or neoplastic lesion of the cervix. Therefore, 79 cases were classified as positive (CIN2+) and 45 as negative (<CIN2). The three specimens (urine, vaginal self-collected and physician collected samples) for all women were submitted to Cobas® 4800 HPV e OncoE6^™^ Cervical Test. S1 Table shows the age, histology and HPV DNA and HPV E6 results of all women enrolled in the study.

### High-risk HPV DNA results (Cobas® 4800 HPV test)

Among the HPV positive cases for any hr-HPV of the physician collected samples (cervical samples), HPV16 was the most frequent type detected (42/82; 51.2%), followed by others hr-HPV (40/82; 48.8%), and HPV18 (10/82; 12.2%). In self-collected vaginal samples, HPV16 DNA was the most detected type (41/81; 50.6%), as well as in the urine samples (36/62; 58.1%). Co-infection rates on cervical sample was 12.2%, whereas in vaginal self-collected samples was 14.8% and in urine samples 14.5%. No case of HPV16 and HPV18 co-infection was detected.

Using the cervical sample collected by the physician as the gold standard, the positivity rate of HPV-DNA was similar to the obtained by the vaginal self-collection (respectively 66.1% vs. 65.3%, p = 1.00), nevertheless it was significantly higher than the urine samples (respectively 66.1% vs. 50.0%, p<0.01) (Table 1).

**Table 1:** Comparison of HPV-DNA test results according to biological specimen origin.

		Cervical sample (physician collection)			p- valor (*)
		Positive	Negative	Total	
**Vaginal self-collection**	Positive	78	3	**81 (65.3%)**	1,00
	Negative	4	39	**43 (34.7%)**	
	**Total**	**82 (66.1%)**	**42 (33.9%)**	124 (100.0%)	
**Urine**	Positive	61	1	**62 (50.0%)**	<0,01
	Negative	21	41	**62 (50.0%)**	
	**Total**	**82 (66.1%)**	**42 (33.9%)**	124 (100.0%)	

reference group: cervical sample

(*)McNemar test

### HPV16/18-E6 results (Onco*E6*^™^ cervical test)

Thirty-eight physician collected specimens were positive on HPV16/18-E6 test, being 31 positive for HPV16-E6 and 7 positive for HPV18-E6. Among the self-collected vaginal samples, 20 were positive for HPV16-E6 and 5 for HPV18-E6. In urine samples, HPV16-E6 was detected in 22 specimens and HPV18-E6 in 4.

Using the cervical sample collected by the physician as the reference, the positivity rate of HPV16/18-E6 test was significantly higher than the vaginal self-collection (respectively 30.6% vs. 20.2%; p<0.01) and the urine (respectively 30.6% vs. 21.0%, p<0.01) (Table 2).

**Table 2:** Comparison of HPV16/18-E6 test results according to biological specimen origin.

		Cervical sample (physician collection)			p- valor (*)
		Positive	Negative	Total	
**Vaginal self-collection**	Positive	23	2	**25 (202%)**	<0,01
	Negative	15	84	**99 (79.8%)**	
	**Total**	**38 (30.6%)**	**86 (69.4%)**	124 (100.0%)	
**Urine**	Positive	22	4	**26 (21.0%)**	0,01
	Negative	16	82	**98 (79.0%)**	
	**Total**	**38 (30.6%)**	**86 (69.4%)**	124 (100.0%)	

reference group: cervical sample

(*)McNemar test

**Fig 1:**
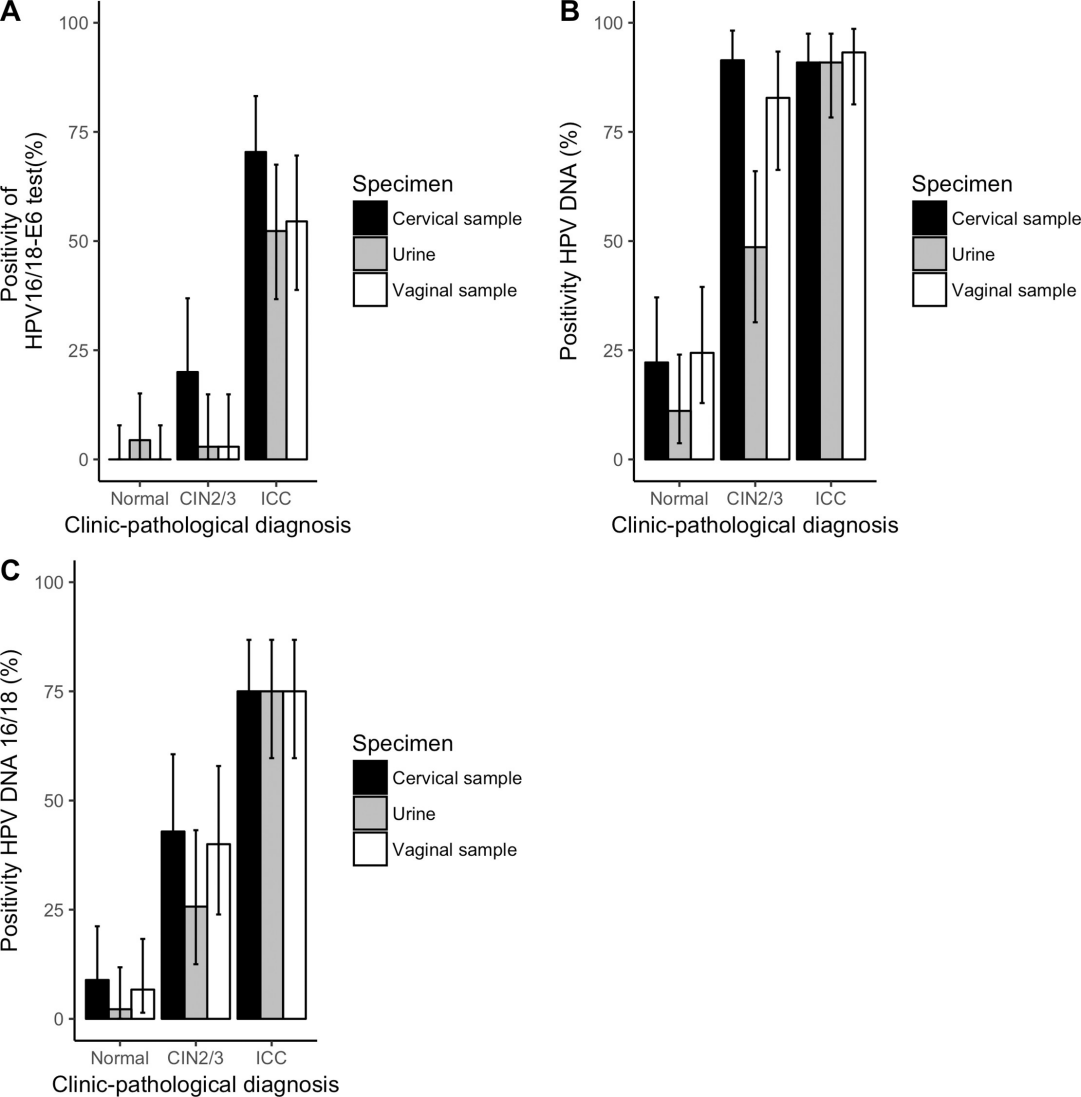
Positivity rates of **(A)** HPV16/18-E6 test, **(B)** HPV DNA test for any hr-HPV, **(C)** HPV16 or 18 DNA test with 95% confidence interval in paired vaginal self-collection, cervical physician collection and urine specimens. CIN, cervical intraepithelial neoplasia; ICC, invasive cervical cancer.

Fig 1 shows the positivity rates of (A) HPV16/18-E6 test, (B) HPV DNA test for any hr-HPV, (C) HPV16 or 18 DNA test with 95% confidence interval in paired vaginal self-collection, cervical physician collection and urine specimens. Concerning the HPV16/18-E6 test (Fig 1A), similar positivity rates were observed in the vaginal specimen (self-collection) and in the urine, both in the group of women diagnosed with microscopic lesions (CIN2/3) and in the group of invasive cervical carcinoma. However, the detection rate of the E6 oncoprotein was higher in specimens collected directly from the cervix by the physician. Regarding the positivity of HPV16/18-E6 specifically in urine it was significantly higher in the group of women with invasive carcinoma compared to the other groups (<CIN2 and CIN2/3). No HPV16/18-E6 positive result was obtained in the CIN2 group (0/9) in the three type of specimens. In CIN3 group, the HPV16/18-E6 was positive in 26.9% (7/26) of the cervical samples and in 3.8% (1/26) of both, vaginal and urine samples. There was no significant difference in urine HPV16/18-E6 positivity between the group of women without cervical injury and those diagnosed with high-grade precursor lesion (CIN2/3).

In the HPV DNA test (Cobas), rates were progressively higher according to the severity of the diagnosis (Fig 1B and 1C). High-grade precursor lesions (CIN2/3) had similar HPV DNA-positivity rate in cervical and vaginal specimens, and lower in urine specimens. For invasive lesions, there was no statistically significant difference according to the origin of the biological sample (cervix, vagina and urine).

### Comparative analysis between the HPV16/18-E6 test and the HPV16/18-DNA test

The E6-HPV test used in this study is specific for HPV16 and HPV18 E6 oncoprotein, then comparative analysis with the HPV DNA test was performed only for these specific HPV types. Table 3 shows the comparison of the positivity rate of the methods and agreement analysis.

**Table 3:** Comparison between HPV-DNA and HPV16/18-E6 test results for types 16 and 18 according to sample origin.

Specimen	HPV-DNA (16/18)	HPV16/18-E6			Kappa	p-value(*)
		Positive	Negative	Total		
**Cervical sample (physician collection)**	Positive	38	14	**52 (41.9%)**	0,76(**)	<0,01
	Negative	0	72	**72 (58.1%)**		
	**Total**	**38 (30.6%)**	**86 (69,4%)**	124 (100%)		
**Vaginal self-collection**	Positive	25	25	**50 (40,7%)**	0,54 (**)	<0,01
	Negative	0	73	**73 (59,3%)**		
	**Total**	**25 (20.3%)**	**98 (79,7%)**	123 (100%)		
**Urine**	Positive	23	20	**43 (34.7%)**	0,55 (**)	<0,01
	Negative	3	78	**81 (65.3%)**		
	**Total**	**26 (21.0%)**	**98 (79,0%)**	124 (100%)		

(*) Teste de McNemar

(**) p-valor < 0,05 (Kappa)

The HPV16/18-E6 positivity rate was significantly lower (<0.01) than the HPV DNA positivity rate for HPV types 16 and 18 when the analysis was stratified by specimen type (cervical, vaginal and urine) (Table 3). Comparison of the HPV16/18-E6 test with the HPV DNA test showed moderate agreement in the urine and vaginal samples (self-collection), and moderate to strong agreement in the cervical sample (physician-collection).

### Clinical accuracy of HPV16/18-E6 and HPV-DNA tests

Accuracy analyzes of the HPV16/18-E6 and HPV-DNA tests were performed stratified for CIN2, CIN3 and invasive carcinomas, and are shown in Tables 4 and 5, respectively.

**Table 4:** Clinical sensitivity and specificity (95% confidence interval) of HPV16/18-E6 test and HPV-DNA test to detection of high-grade cervical intraepithelial neoplasia (CIN2/3).

Specimen	Indicator	HVP16/18-E6 test	HPV-DNA test (HPV16/18)	HPV-DNA test (14 hr-HPV types)
**CIN2 cases (N = 9)**				
**Cervical sample (physician collection)**	Sensitivity	0.00 (0.00–0.34)	0.22 (0.03–0.60)	0.78 (0.40–0.97)
	Specificity	1.00 (0.92–1.00)	0.91 (0.79–0.98)	0.78 (0.63–0.89)
**Vaginal self-collection**	Sensitivity	0.00 (0.00–0.34)	0.22 (0.03–0.60)	0.67 (0.30–0.93)
	Specificity	1.00 (0.92–1.00)	0.93 (0.82–0.99)	0.76 (0.60–0.87)
**Urine**	Sensitivity	0.00 (0.00–0.34)	0.22 (0.03–0.60)	0.44 (0.14–0.79)
	Specificity	0.96 (0.85–0.99)	0.98 (0.88–1.00)	0,89 (0.76–0.96)
**CIN3 cases (N = 26)**				
**Cervical sample (physician collection)**	Sensitivity	0.27 (0.12–0.48)	0.50 (0.30–0.70)	0.96 (0.80–1.00)
	Specificity	1.00 (0.93–1.00)	0.89 (0.77–0.96)	0.69 (0.54–0.80)
**Vaginal self-collection**	Sensitivity	0.04 (0.00–0.20)	0.46 (0.27–0.67)	0.88 (0.70–0.98)
	Specificity	1.00 (0.93–1.00)	0.91 (0.80–0.97)	0.69 (0.54–0.80)
**Urine**	Sensitivity	0.04 (0.00–0.20)	0.27 (0.12–0.48)	0.50 (0.30–0.70)
	Specificity	0.96 (0.87–1.00)	0.94 (0.85–0.89)	0.83 (0.71–0.92)

**Table 5:** Clinical sensitivity and specificity (95% confidence interval) of HPV16/18-E6 test and HPV-DNA test to invasive cervical cancer detection.

Specimen	Indicator	HVP16/18-E6 test	HPV-DNA test (HPV16/18)	HPV-DNA test (14 hr-HPV types)
**Cervical sample (physician collection)**	Sensitivity	0.70 (0.55–0.83)	0.75 (0.60–0.87)	0.91 (0.78–0.97)
	Specificity	0.91 (0.83–0.96)	0.76 (0.65–0.85)	0.47 (0.36–0.59)
**Vaginal self-collection**	Sensitivity	0.55 (0.39–0.70)	0.77 (0.61–0.88)	0.93 (0.81–0.99)
	Specificity	0.99 (0.93–1.00)	0.79 (0.68–0.87)	0.50 (0.39–0.61)
**Urine**	Sensitivity	0.52 (0.37–0.68)	0.75 (0.60–0.87)	0.91 (0.78–0.97)
	Specificity	0.96 (0.89–0.99)	0.88 (0.78–0.94)	0.72 (0.61–0.82)

The HPV16/18-E6 HPV test had a significantly lower sensitivity rate than the HPV-DNA test for both CIN2 and CIN3 detection, regardless of the type of specimen (cervical, vaginal or urine). On the other hand, it presented higher specificity rate than the HPV-DNA test.

Concerning the diagnosis of invasive cervical carcinoma, the HPV16/18-E6 test had a lower sensitivity rate than the HPV-DNA test in all samples (cervical, vaginal and urine), but achieved higher specificity rates. The only situation in which the HPV16/18-E6 test had similar sensitivity to the HPV-DNA test was only when HPV16 and 18 HPV DNA were evaluated.

## Discussion

Cervical cancer remains an urgent problem of public health, especially in low- and middle-income countries [1]; reasons are poor uptake of screening programs [6, 7], lack of adequate programs, lack of appropriate screening technologies, and lack of funds to promote such programs, to just name a few. Several new technologies have emerged during the past few years to address the quest for appropriate screening technologies. One general approach focuses on application of biomarkers that promise to be highly specific for true cervical malignancy, thus reducing costly over treatment. Another recent development is sample self-collection, in an effort to mitigate infrastructure and cultural barriers. In this study, we evaluated the HPV E6 oncoprotein as such a biomarker for cervical neoplasia, in conjunction with different ways of specimen collection. In particular, we were interested in the question whether women with histology confirmed precursor lesions or cervical cancer would present E6 oncoprotein in samples of their urine. This is the first study reporting detection of HPV E6 oncoproteins from urine samples via the use of a lateral flow (“strip test”) immunochromatographic method on women with cervical cancer. In more detail, we applied the Onco***E6***^™^ Cervical Test and the Cobas® 4800 HPV Test to physician collected cervical samples, to self-collected vaginal samples, and to urine samples. Our results showed that the E6 oncoproteins of HPV16 and/or 18 were present in urine specimens from women with cervical cancer and were detected at similar rates as the vaginal self-collected samples. Sensitivity for CIN2/3 detection, however, was lower than what was observed by the HPV DNA test.

The HPV DNA test’s sensitivity for CIN2/3 detection using urine samples in the current study is higher than reported by previous studies that used Cobas HPV test [21, 22], but lower than reported from studies that used the Trovagene platform [24, 26]. The specificity obtained was overall higher than reported from studies that used Trovagene and Cobas HPV tests [22, 24, 26, 27]. The higher sensitivity obtained in our study in comparison with other studies that used Cobas HPV test might be explained by the use of an EDTA preservative solution for our specimens.

The hr-HPV prevalence in cervical samples based on the Cobas HPV test outcome was high due to an intended selective bias: almost 64% of the selected women had CIN2/3 or invasive cervical cancer. The hr-HPV prevalence in the Brazilian population is around 12% [34, 35].

The HPV DNA test exhibits a higher sensitivity for detection of CIN2/3 than the HPV16/18-E6 test; this is true for all three types of specimens; for all types of specimens, however, the specificity was higher for the HPV16/18-E6 test than for the HPV DNA test. This result was expected, partly because Cobas HPV Test detects 14 hr-HPV types and the OncoE6 test detects the E6 oncoprotein of only HPV16 and HPV18; Fifty-seven per cent of the CIN2/3 cases were either HPV negative or positive only for hr-HPV other than HPV16 or 18, while this number decreased to 25% for invasive carcinoma. It has been shown that elevated expression of the E6 oncoprotein is also an indicator of persistence of viral infection [36], thus some HPV16 or 18 DNA positive cases that are HPV16/18-E6 test negative could represent transient infections or cervical intraepithelial neoplasia that will regress. Previously, it has been demonstrated that only a fraction (of at most 50%) of CIN3 lesions will progress to cancer within a 30-year time span [37]; it appears plausible, thus, that only a fraction of CIN3 lesions will have elevated oncoproteins levels, while those CIN3 that lack the ability to progress further also lack elevated levels of the viral oncoproteins.

Using the HPV16/18-E6 test, similar rates of detection of high-grade lesions were seen for the self-collected vaginal samples and the urine samples, and both these sample types had a lower detection rate than the physician-collected sample. Previous studies evaluating the HPV16/18-E6 test with clinician-collected samples showed lower sensitivity and specificity for detection of high-grade lesion than we observed in our study. Zhao et al. conducted a study in rural areas of China using the OncoE6 Test and obtained 42.4% of sensitivity and 99.1% of specificity with CIN2+ as an endpoint [38]. In a study by Mariano et al., performed in Barretos (rural Brazil), 49.6% of sensitivity and 91.8% of specificity were achieved [31], quite similar to the outcome of the here reported study. With cervical physician collected specimens, no positive outcome (“false positives”) was seen via the HPV16/18-E6 test in women without malignant pathology by histology. Two urine specimens tested positive on the E6 test while testing HPV negative via Cobas and testing negative also via E6 test on the corresponding physician and vaginal self-collected specimens. One of the cases turned out to be an invasive carcinoma (HPV16 E6 positive via Onco***E6***^™^ Cervical Test), and the other was a case of normal cytology testing positive for HPV18 E6.

Given the importance of improving the adherence to cervical cancer screening, researchers are trying to validate sampling options that are more acceptable to the target population, like the self-collected vaginal samples and urine specimens that can be collected at home [11–17]. In France, for example, women who had not attended the invitation for a PAP smear were invited to perform a home-based urine sampling procedure for HPV DNA testing; 13.7% of women returned a urine sample. This rate of participation is comparable to that obtained by the reminder mailing of the national organized screening [17].

Urine sampling is a non-invasive self-collection method, and, it is highly accepted by women [26, 39]. It has repeatedly been shown, that HPV can be detected (via DNA) in the urine of women who have cervical/vaginal HPV. This is explained most likely by the fact that exfoliated cervical epithelial cells are found in the vagina, and that current testing methods for HPV testing (so for example the Cobas PCR HPV test) have a very high analytical sensitivity. Some studies evaluating the urine-based HPV DNA detection had variable results [18–25], and this may be due mainly to a lack of standardization (first void versus initial stream versus random urine) and other testing protocols parameters [40]. The rationale behind the use of urine specimens towards DNA based HPV detection is that superficial cell layers exfoliated and mixed with secretions of the vagina and uterine cervix, flows through the vagina and thus fiablly localize at the ostium of the urethra, before being eventually flushed away with the urine flow. Accordingly, the initial stream of urine flow should have a higher concentration of mucus and debris from exfoliated cells [41] than a subsequent urine flow. To date, however, no pattern on the effect of use of the initial void of urine (in the morning) versus urine samples given at any time of the day with regard to analytical sensitivity of HPV detection have been observed [26]. For this study, we hence decided to be agnostic with regard to the day time of sample collection.

This study was developed as a proof of concept for detecting the HPV E6 oncoprotein in urine samples and was submitted to a bias on the samples’ selection because we preferred to work with more invasive/microinvasive lesions than non-invasive cases which will increase our changes of detecting the E6 oncoprotein in the urine samples. The true value of detecting E6 oncoprotein for cervical cancer screening purposes should be addressed in other studies enrolling a large number of specimens with an appropriate sample size calculation.

## Conclusions

This study shows that the detection of HPV16/18 E6 oncoprotein is feasible in urine samples of invasive lesions. While it may seem surprising and encouraging that a majority of cervical cancer related to HPV16 and/or 18 could be readily detected via E6 oncoprotein present in the urine, further protocol development and standardization to achieve a clinical sensitivity for CIN2/3 detection close to what can be achieved for invasive lesions using the physician collected cervical is needed. We believe that the outcome of our study warrants such further development. Large population-based studies with more HPV types included are essential in future.

## Supporting information

S1 Table Age, histology and HPV DNA and HPV E6 status of all women enrolled in the study. (XLS)
